# Internet-delivered emotional awareness and expression therapy for somatic symptom disorder: one year follow-up

**DOI:** 10.3389/fpsyt.2024.1505318

**Published:** 2025-01-03

**Authors:** Henrik Hallberg, Daniel Maroti, Mark A. Lumley, Robert Johansson

**Affiliations:** ^1^ Department of Psychology, Stockholm University, Stockholm, Sweden; ^2^ Department of Psychology, Wayne State University, Detroit, MI, United States

**Keywords:** somatic symptom and related disorders (SSRDs), functional somatic disorder (FSD), emotional awareness and expression therapy, internet delivered psychological treatments, guided self help

## Abstract

**Objective:**

We examined whether the treatment effects from a previous RCT of Internet-delivered Emotional Awareness and Expression Therapy (I-EAET) for somatic symptom disorder were maintained 12 months after treatment.

**Method:**

12-month assessments of self-reported somatic symptoms, pain severity, and several secondary outcomes were compared with baseline and post-treatment levels within the I-EAET condition only, given that the waitlist control condition had already received treatment. Twenty-eight out of the original 37 participants (76%) in the I-EAET condition provided follow-up data.

**Results:**

The beneficial effects of I-EAET on somatic symptoms observed at post-treatment were maintained at the 12-month follow-up (d = -0.22, 95% CI: -0.72 to 0.28), as well as for pain intensity (d = -0.02, 95% CI: -0.52 to 0.48). From pre-treatment to 12-month follow-up, there was a medium effect on somatic symptoms (*d* = 0.74, 95% CI 0.23 to 1.24), and a small, non-significant effect for pain intensity (*d* = 0.43, 95% CI -0.06 to 0.93). Response rates (at least 50% symptom reduction) at 12-month follow-up were 25% for somatic symptoms, and 12% for pain intensity.

**Conclusion:**

I-EAET seems to have positive long-term effects for somatic symptom disorder. Larger studies with controls and comparisons to other treatments are needed.

## 1 Introduction

Somatic symptom disorder (SSD) is defined as having one or more chronic somatic symptoms that are distressing or disruptive of daily life, as indicated by disproportionate and dysfunctional cognitive, emotional, and behavioral responses. For example, someone with SSD may present with chronic pain, bowel disruptions, or fatigue that is accompanied by anxiety, catastrophic thinking, and behavioral avoidance. The prevalence of SSD is 5 to 7% in the general population (1) and up to 17% in primary care (2). SSD tends to be chronic—up to 90% of patients have symptoms beyond 5 years (3)—and disruptive—the somatic symptoms in people with SSD are associated with psychiatric comorbidity and functional disability such as work impairment or early retirement (4).

One promising treatment for SSD is Emotional Awareness and Expression Therapy (EAET) (5). EAET includes psychoeducation about the central nervous system control of pain and other somatic symptoms, exploration of links between somatic symptoms and unresolved trauma, emotional processing of trauma and conflict, reattribution of somatic symptoms to emotional and brain-based processes, the development of a self-soothing capacity using self-compassion, and encouragement to improve adaptive interpersonal communication. EAET has been tested in randomized controlled trials (RCTs) for people with fibromyalgia (6), irritable bowel syndrome (7), urogenital pain (8), medically unexplained symptoms (9), and musculoskeletal pain (10, 11) and found to be superior to treatment as usual, education controls, and even cognitive behavioral therapy (CBT) (6, 10, 11). In our original RCT, which forms the basis for this follow-up study, an Internet-delivered version of EAET (I-EAET) was superior to a waitlist control in reducing somatic symptoms and pain intensity at post-treatment (small to medium effect size). It also showed superiority for somatic symptoms at the 4-month follow-up, response rates for somatic symptoms at follow-up, and also for depression and anxiety at post-treatment, but not at follow-up. (12). In other clinical trials of EAET, treatment effects have been maintained at short-term follow-up assessments, ranging from 2 to 6 months, but the effects of EAET in any modality after 6 months have not been studied. The purpose of the current secondary analyses was to examine whether the effects of I-EAET at post-treatment of our earlier trial were maintained at 12-month follow-up (12). Because the waitlist condition was offered I-EAET after the 4-month follow-up, the current analyses are within the I-EAET condition only and do not include a control condition.

## 2 Materials and methods

### 2.1 Study design

The original RCT for people with persistent somatic symptoms (N = 74) compared I-EAET (n = 37) to a waitlist control (n = 37). All participants were diagnosed with SSD, with a physician ruling out diseases (e.g., cancer or rheumatoid arthritis) as the cause of the somatic symptoms. I-EAET lasted for 10 weeks and was provided through a secure web-platform (KI eHealth Core Facility) used by Karolinska Institute. The two study arms were compared at post-treatment and 4-month follow-up, at which point, the waitlist control participants were provided I-EAET. The recruitment, screening, randomization, measures, and intervention are fully described in (12). The trial was pre-registered at ClinicalTrials.gov (NCT04751825). Informed consent was given while registering for the study and included follow-up measurements. In this follow-up study, the 37 participants from the I-EAET condition were contacted 1 year after treatment termination to assess their primary and secondary outcomes.

### 2.2 Measures and statistical analysis

Two primary outcome measures were assessed at pre-treatment, post-treatment, as well as at 4-month and 12-month follow-ups. Somatic symptom severity during the last week was assessed with the Patient Health Questionnaire-15 (PHQ-15) (13, 14); items are rated 0 (“not bothered at all”), 1 (“bothered a little”), or 2 (“bothered a lot”) and summed. Pain intensity was assessed with four items from the Brief Pain Inventory (BPI-4): worst, least, and average pain over the past week, and current pain. Items are rated from 0 (“no pain”) to 10 (“worst imaginable pain”) and averaged (15).

Three secondary outcome measures were assessed at the same timepoints: the Patient Health Questionnaire-9 (PHQ-9) for depression (16), Generalized Anxiety Disorder-7 (GAD-7) for anxiety (17), and the Post Traumatic Stress Disorder Checklist (PCL-5) for trauma symptoms (18).

All 37 participants were included in the effect size analyses, applying intention to treat. The online software Psychometrica (19) was used for all effect size analyses. Welch tests, with SPSS 26 (20), were used for significance testing, comparing pre- and post-treatment to 12-month follow-up. Effect sizes in the range of 0.20–0.49 were considered small, 0.50–0.79 medium, and over 0.80 large (21).

Substantial response to treatment was defined as 50% or greater symptom reduction from pre- to post-treatment and to follow-up for each of the two primary outcomes (PHQ-15/somatic symptoms and BPI-4/pain intensity). Chi-square tests were conducted to examine condition differences in prevalence of substantial responders, using Psychometrica (19). Participants not providing follow-up data were excluded from these responder analyses.

The original trial registration noted that we would assess sleep-related outcomes (Epworth Sleepiness Scale, Insomnia Severity Index) and functional impairment (Sheehan Disability Scale); however, these measures were not included at the 12-month follow-up, due to research error. See Maroti et al. (12) for further elaboration.

## 3 Results

### 3.1 Participants, adherence, attrition, and missing data

The original sample of 37 participants was 81% female, age 23-64. Regarding psychiatric comorbidity, the most common diagnosis was depression—almost 70% had recurrent or ongoing depression. There was a range of somatic diagnoses, with IBS (25%) or migraine/severe headache (20%) as the most common. For details, see Maroti et al. (2022) (11).

All 10 I-EAET modules were completed by 83.8% of the 37 participants; the mean number of completed modules was 8.8. Attrition was very low; only 2 I-EAET participants (5.4%) terminated participation, and 34 of the 37 (92%) provided post-treatment data. At 12-month follow-up, 28 of 37 (76%) provided data for the two primary outcomes, and 26 of 37 (70%) provided data for three secondary outcomes.

### 3.2 Primary and secondary outcomes

Results are displayed in **Table 1**. We compared within-condition effects from pre-treatment (baseline) to 12-month follow-up. Somatic symptoms were significantly reduced with a medium effect (*d =* 0.74, 95% CI 0.23–1.24, *p* = 0.005). Pain intensity had a small effect, but it was not significant. (*d* = 0.43, 95% CI: -0.06–0.93, *p* = 0.09). Comparing post-treatment assessment to 12-month follow-up, there was no significant change in somatic symptoms (*d =* -0.22, 95% CI: -0.72–0.28, *p* = 0.40), or pain intensity (*d = -*0.02, 95% CI: -0.52–0.48, *p* = 0.96).

**Table 1:** Means, SDs, and effect sizes (Cohen’s *d*) for different outcome measures within the I-EAET condition only.

		Mean (SD)			Effect size *d* (95% CI), within-condition	
Primary measures	Pre (*n* = 37)	Post (*n* = 34)	4-month FU (*n* = 33)	12-month FU (*n*= 28)	Pre vs 12-month FU	Post vs 12-month FU
*Somatic symptoms* Patient Health Questionnaire (PHQ-15)	12.1 (3.92)	8.18 (3.52)	8.73 (3.82)	9.04 (4.38)	*d* = 0.74** [0.24, 1.25] *p* = 0.005	*d* = -0.22 [-0.72, 0.28] *p* = 0.405
*Pain intensity* Brief Pain Inventory (BPI-4)	4.28 (1.70)	3.49 (1.97)	3.65 (1.94)	3.52 (1.82)	*d* = 0.43 [-0.06, 0.93] *p* = 0.09	*d* = -0.02 [-0.52, 0.48] *p* = 0.959
Secondary measures	(*n* = 37)	(*n* = 34)	(*n* = 32)	(*n* = 26)		
*Depression* Patient Health Questionnaire (PHQ-9)	10.0 (5.64)	7.65 (4.56)	7.42 (6.25)	9.04 (5.79)	*d* = 0.17 [-0.33, 0.67] *p* = 0.503	*d* = -0.27 [-0.78, 0.24] *p* = 0.318
*Anxiety* Generalized Anxiety Disorder (GAD-7)	6.58 (5.65)	5.53 (4.56)	5.64 (4.94)	6.58 (4.92)	*d* = 0.00 [-0.50, 0.50] *p* = 0.978	*d* = -0.22 [-0.74, 0.29] *p* = 0.403
*PTSD symptoms* The Posttraumatic Stress Disorder Checklist for *DSM-5* (PCL-5)	16.8 (15.7)	20.6 (18.6)	22.2 (16.9)	22.1 (20.5)	*d* = -0.30 [-0.80, 0.21] *p* = 0.265	*d* = -0.08 [-0.59, 0.43] *p* = 0.848

**p<0.01. *p<0.05.

There were no significant changes in any of the secondary outcomes from pre-treatment to 12-month follow-up or from post-treatment to 12-month follow-up.

### 3.3 Responder analyses

For somatic symptoms (PHQ-15), 7 of 34 (21%) of I-EAET participants were classified as responders at post-treatment, and 7 of 28 (25%) were at follow-up; these percentages did not differ, *X* ^2^ (1, *N* = 34) = 0.17, *p* = 0.68. For pain intensity, 8 of 34 (24%) participants were classified as responders at post-treatment, and 4 of 28 (12%) were responders at 12-month follow-up; these percentages did not differ, *X* ^2^ (1, *N* = 34) = 0.84, *p* = 0.36. We did additional analyses to explore whether the 6 participants who provided post data but not 12-month follow-up data, differed from the 28 participants who did provide 12-month follow-up data. See **Table 2**. Statistical significance was tested with Welch tests at both primary measures, and Chi^2^-tests for response rates. All p-values were larger than 0.05, indicating no statistically significant differences at the main outcomes between the participants providing 12-month follow-up data and the ones who did not.

**Table 2:** Means, SDs, and significance tests comparing participants providing/not providing data at 12-month follow-up.

Outcome measure	Complete data at 12-month follow-up (n=28)	Missing data at 12-month follow-up (n=6)	*p*-value
Somatic symptoms/PHQ-15 pre (SD)	11.82 (4.06)	12.83 (3.87)	*p*=0.58
PHQ-15 post (SD)	8.32 (3.64)	7.50 (3.08)	*p*=0.58
Pain severity/BPI-4 pre (SD)	4.32 (1.50)	3.88 (2.02)	*p*=0.63
BPI-4 post (SD)	3.50 (2.02)	3.46 (1.89)	*p*=0.96
Response rate PHQ-15 post	21% (6/28)	17% (1/6)	*p*=0.82
Response rate BPI-4 Post	25% (7/28)	17% (1/6)	*p*=0.66

## 4 Discussion

This is the first study to assess whether somatic symptom reduction at the end of EAET was maintained at 12-month follow-up. The significant post-treatment reductions in somatic symptoms for our participants who received I-EAET were largely maintained 12 months later, with a medium effect size, and the percentage of responders (50% or more reduction in somatic symptoms) was also maintained. The small trend toward increased somatic symptoms between post-treatment and 12-month follow-up was non-significant. The effect on pain severity at 12-month follow-up, however, was no longer significant. On secondary measures, treatment effects were originally not significant at post-treatment, and the 12-month follow-up reflected a return of anxiety and depression. The average levels of depression and anxiety at pre-treatment were, however, only in the mild range, and post-traumatic symptoms were in the subclinical range; these low levels on these measures likely limited improvement.

These results provide preliminary evidence that I-EAET has positive effects 12 months after treatment, at least on reducing somatic symptoms. One might compare these results with those of internet-cognitive behavioral therapy (I-CBT) for similar populations. The effects of I-CBT on pain, compared to controls at post-treatment, are small (22), and the few studies of I-CBT that have 12-month follow-ups have inconsistent results—two studies showed maintained benefits within-condition (23, 24), the third study had a small effect size within-group but not compared to the control condition (25). There is evidence for deterioration over time for people with chronic pain who are untreated (3), whereas the current study found that the positive effect of I-EAET on somatic symptoms was largely maintained. However, we still know too little about what works for people with somatic symptoms in the long-term, and I-EAET and I-CBT have never been directly compared in a trial.

This study has several limitations. Most importantly, these analyses at 12-month follow-up with only within-condition were not compared to a control condition because the original waitlist control participants were offered I-EAET 4 months post-treatment. This lack of a control group precludes concluding that the treatment itself led to the maintenance of improvements. We also do not know what other treatments participants might have obtained over the follow-up period, although the availability of effective treatments for SSD is limited. Moreover, it remains unclear whether the effects of I-EAET observed at the 12-month follow-up are generalizable to other forms of EAET, such as face-to-face individual therapy or group formats. Further, the small sample size limits the statistical power of the study to detect significant effects. Future studies should include control conditions and direct comparisons to other active treatments (e.g., internet-CBT) and have larger samples.

In conclusion, this study provides preliminary evidence that I-EAET has positive effects on somatic symptoms (although not other measures) for people with SSD at 12-month follow-up.

## Data Availability

The data that support the findings of this study are available from the corresponding author upon reasonable request.
